# Early Müller Glial Activation and Retinal Ganglion Cell Synaptic Dysfunction in APP/PS1 Mice

**DOI:** 10.3390/cells15090801

**Published:** 2026-04-28

**Authors:** Yuyan Zhou, Guibo Qi, Haoyang Zhou, Pifang Gong, Zhenru Wang, Xuan Song, Cheng Tian, Haixiang Wu, Song Qin

**Affiliations:** 1 Eye Institute and Department of Ophthalmology, Eye & ENT Hospital, Fudan University, Shanghai 200031, China; 18301050100@fudan.edu.cn; 2 Department of Anatomy, Histology and Embryology, School of Basic Medical Sciences, State Key Laboratory of Brain Function and Disorders, Fudan University, Shanghai 200032, China; 20211010033@fudan.edu.cn (G.Q.);; 3 Department of Hand Surgery, Huashan Hospital, Fudan University, Shanghai 200040, China; 19301020031@fudan.edu.cn

**Keywords:** Alzheimer’s disease, Müller glia, glutamine synthetase, glial morphology, retinal ganglion cells

## Abstract

**Highlights:**

**What are the main findings?**

- Early synaptic dysfunction of retinal ganglion cells occurs in APP/PS1 mice prior to overt neuronal loss and in the absence of retinal Aβ plaques.
- Müller glia undergo pronounced activation and remodeling accompanied by altered glutamine synthetase activity and disrupted aquaporin-4 polarity.

**What are the implications of the main findings?**

- Retinal dysfunction in Alzheimer’s disease may arise from early synaptic vulnerability rather than local amyloid plaque deposition.
- Müller glial remodeling may represent an early indicator and potential modulator of neurodegenerative processes in the retina.

**Abstract:**

Alzheimer’s disease (AD) is increasingly recognized as a multisystem neurodegenerative disorder in which sensory dysfunction accompanies cognitive decline. As an accessible extension of the central nervous system, the retina provides a valuable window for investigating early neurodegenerative processes; however, the cellular mechanisms underlying AD-associated retinal pathology remain incompletely understood. Here, using the APP/PS1 mouse model, we systematically examined structural, functional, and glial alterations in the retina across disease stages. Despite robust age-dependent amyloid plaque accumulation in visual-related brain regions, no plaque-like β-amyloid (Aβ) deposits were detected in the retina even at advanced ages. Nevertheless, young APP/PS1 mice exhibited early thinning of inner retinal layers, impaired retinal electrophysiological responses, and reduced excitatory synaptic inputs to retinal ganglion cells (RGCs), preceding overt neuronal loss. These neuronal changes were accompanied by pronounced Müller glial activation, characterized by upregulation of gliosis markers and extensive morphological remodeling. Functional analyses further revealed dynamic alterations in glial homeostasis, including early elevation followed by age-dependent decline of glutamine synthetase activity, together with increased expression and disrupted perivascular polarity of aquaporin-4. Consistently, transcriptomic profiling of young AD retinas identified coordinated dysregulation of genes involved in amino acid metabolism, transport, and oxidative stress responses. Together, our findings identify Müller glial remodeling as an early feature of AD-associated retinal pathology that coincides with synaptic vulnerability of RGCs and occurs independently of local Aβ plaque deposition, highlighting retinal glia as potential early indicators and modulators of neurodegeneration.

## 1. Introduction

Alzheimer’s disease (AD) is the most prevalent neurodegenerative disorder and is clinically characterized by progressive memory loss and cognitive decline [1]. Beyond cognitive impairment, accumulating evidence indicates that sensory dysfunction frequently accompanies AD and may represent an integral component of disease progression rather than a coincidental comorbidity [2,3]. The pathological hallmarks of AD, amyloid beta (Aβ) plaques and neurofibrillary tangles, are thought to drive neuronal dysfunction and reactive gliosis throughout the central nervous system (CNS) [4,5,6]. Given the long preclinical phase of AD and the irreversible nature of neuronal loss, identifying early pathological changes and accessible biomarkers is critical for timely diagnosis and therapeutic intervention [7].

The retina, embryologically derived from the diencephalon, shares many cellular and molecular characteristics with the brain and thus provides a unique system for studying CNS neurodegeneration [8]. Importantly, retinal imaging is non-invasive, cost-effective, and widely accessible, with technologies such as optical coherence tomography (OCT) enabling high-resolution structural analysis in vivo [9,10]. Consistent with this concept, multiple clinical studies have reported thinning of the retinal nerve fiber layer and the ganglion cell–inner plexiform layer in AD patients [11,12], suggesting that retinal alterations may reflect early neurodegenerative processes associated with the disease.

Retinal ganglion cells (RGCs) serve as the principal output neurons of the retina, transmitting visual information to the brain through the optic nerve. In addition to OCT-detected retinal thinning, degeneration of RGCs in AD is supported by observations of neuronal loss in both animal models and human retinal tissue [13]. However, whether synaptic alterations in RGCs occur prior to overt neuronal degeneration in AD remains largely unexplored. In other neurodegenerative conditions such as glaucoma, synapse disassembly represents an early pathogenic event that precedes dendritic retraction, axonal degeneration, and neuronal loss of RGCs [14,15], raising the possibility that early synaptic vulnerability may also contribute to retinal pathology in AD.

Müller glia span the entire thickness of the neural retina and play essential roles in maintaining retinal homeostasis by regulating neurotransmitter recycling, metabolic support, ion balance, and structural stability [16]. Transcriptomic analyses of AD mouse retinas have identified Müller glia as one of the most responsive cell populations relative to other retinal cell types [17]. In response to stress or injury, Müller glia undergo reactive gliosis characterized by upregulation of intermediate filament proteins such as glial fibrillary acidic protein (GFAP) and vimentin [18]. In AD models, increased GFAP expression in Müller glia has been reported [19], whereas analyses of postmortem human retinas have produced inconsistent findings, including reduced GFAP and glutamine synthetase (GS) immunoreactivity [20]. GS, a Müller glia-enriched enzyme in the retina, catalyzes the conversion of glutamate and ammonia to glutamine and plays a critical role in preventing excitotoxicity [21]. In addition, altered polarity of the glial water channel aquaporin-4 (AQP4) has been reported in both AD mouse and human retinas [22], suggesting disrupted glial homeostasis. Despite these observations, the functional significance and temporal dynamics of Müller glial alterations in the AD retina remain poorly understood.

We previously characterized reactive astrogliosis in the cortex and sex-specific neuropathology in the hypothalamus using the APP/PS1 transgenic mouse model [23,24], which develops cerebral amyloidosis beginning at 6–8 weeks of age [25]. However, whether pathological processes in the retina parallel or diverge from those observed in the brain remains unclear.

In the present study, we investigated retinal pathology in APP/PS1 mice at both early and late disease stages. We examined Aβ deposition along the visual pathway, assessed retinal structure and function using OCT and electroretinography, and evaluated RGC integrity together with Müller glial activation by immunohistochemical and biochemical analyses. Using genetic tracing in Glul-CreERT2-based reporter mice, we further characterized morphological remodeling of Müller glia during disease progression. Finally, transcriptomic profiling of young AD retinas revealed coordinated alterations in amino acid metabolism and transport pathways. Together, these analyses provide a comprehensive view of early retinal dysfunction and Müller glial remodeling in AD.

## 2. Materials and Methods

### 2.1. Animals

The following transgenic mouse lines were used: APP/PS1 mice (Thy1–APPKM670/671NL; Thy1–PS1L166P) [25], Glul-CreERT2-EGFP mice, and Ai14 reporter mice (Rosa26-tdTomato; JAX stock #007914). These lines were intercrossed over multiple generations to generate APP/PS1; Glul-CreERT2; Ai14 mice. Genotyping was performed as previously described [24]. All animals were housed in a controlled environment under a 12 h light/12 h dark cycle with ad libitum access to food and water. Mice aged 4 months and 14 months were defined as the young and old groups, respectively. All experimental procedures were approved by the Animal Care and Use Committee of Shanghai Medical College, Fudan University.

### 2.2. Optical Coherence Tomography and Fundus Photography

The ganglion cell complex (GCC), comprising the RNFL, GCL, and IPL, serves as a reliable indicator of RGC integrity [26]. OCT and fundus photography were performed on anesthetized mice following pupil dilation with tropicamide eye drops. OCT images were acquired using a spectral-domain OCT system (OPTO, Optoprobe, Pontypridd, Mid Glamorgan, UK). Retinal layer and GCC thicknesses were quantified using the manufacturer’s built-in analysis software (Version 2.0, Optoprobe, Pontypridd, Mid Glamorgan, UK). Fundus images were captured using a retinal imaging system (OPTO-RIS, Optoprobe).

### 2.3. Electroretinogram Recordings

Retinal function was assessed by electroretinography (ERG) and photopic negative response (PhNR) recordings using the Espion Electrophysiology System (Diagnosys LLC, Lowell, MA, USA). Mice were dark-adapted overnight and anesthetized with pupil dilation before recordings. Under dim red illumination, platinum ring electrodes were placed on the corneal surface, with a reference electrode positioned subcutaneously in the frontal region and a ground electrode attached to the tail. Scotopic ERG responses were elicited using a series of flash intensities (0.01, 0.1, 1.0, 3.0, and 10.0 cd·s/m^2^). Five consecutive responses were recorded and averaged at each intensity. Photopic ERG and PhNR recordings were obtained under photopic conditions without dark adaptation using a flash intensity of 10.0 cd·s/m^2^. The initial negative deflection was defined as the a-wave, the subsequent positive peak as the b-wave, and the negative deflection following the b-wave as the PhNR.

### 2.4. Tamoxifen Administration

Tamoxifen was prepared at a concentration of 20 mg/mL by dissolving 200 mg of tamoxifen (T5648, Sigma-Aldrich, St. Louis, MO, USA) in 10 mL of 10% ethanol/corn oil. Mice received daily intraperitoneal injections for 5 consecutive days and were sacrificed 2 days after the final injection.

### 2.5. Retina Tissue Preparation

Mice were deeply anesthetized with pentobarbital sodium and transcardially perfused with phosphate-buffered saline (PBS), followed by 4% paraformaldehyde (PFA). Eyes were enucleated and post-fixed in 4% PFA at 4 °C for 12 h. After fixation, corneas were removed and eyecups were cryoprotected in graded sucrose solutions at 4 °C for 3 days. Retinal tissues were sectioned at 16 μm thickness using a freezing microtome (CM1520, Leica, Nussloch, Germany) and stored at −20 °C until use. For flat-mounted preparations, eyes were post-fixed in 4% PFA at 4 °C for 2 h before retinas were dissected and cut into four radial petals.

### 2.6. Immunofluorescence Staining

Retinal sections or flat-mounted retinas were washed with PBS, permeabilized with 0.1% or 0.3% Triton X-100, and blocked in 5% bovine serum albumin. Samples were incubated with primary antibodies overnight at 4 °C (retinal sections) or for 2 days at 4 °C (flat mounts), followed by incubation with species-specific Alexa Fluor-conjugated secondary antibodies (488, 555, or 647; Jackson ImmunoResearch, West Grove, PA, USA) for 2 h at room temperature. Nuclei were counterstained with Hoechst 33342 (1 μg/mL; Sigma-Aldrich, St. Louis, MO, USA).

Primary antibodies included: 6E10 (mouse, 1:500, 803013, BioLegend, San Diego, CA, USA), Aβ_1–42_ (rabbit, 1:500, ab224275, Abcam, Cambridge, UK), Brn3a (goat, 1:800, sc-31984, Santa Cruz Biotechnology, Dallas, TX, USA), PSD95 (mouse, 1:500, 75-028, NeuroMab, Davis, CA, USA), VGLUT1 (guinea pig, 1:800, OB-PGP101, Oasis, Taizhou, Zhejiang, China), GS (mouse, 1:500, MAB302, MilliporeSigma, Burlington, MA, USA; rabbit, 1:500, G2781, MilliporeSigma), GFAP (mouse, 1:1000, G3893, MilliporeSigma), vimentin (chicken, 1:500, AB5733, MilliporeSigma), AQP4 (mouse, 1:1000, GB15529, Servicebio, Wuhan, Hubei, China), and CD31 (rat, 1:500, 550274, BD Biosciences, San Jose, CA, USA).

### 2.7. Microscopy and Morphological Analysis

Images were acquired using an Olympus FV3000 confocal laser scanning microscope (Olympus, Tokyo, Japan) under identical acquisition settings across experimental groups. Z-stack images were collected at 1.0 μm intervals with 10 optical sections per stack. Image processing and analysis were performed using ImageJ (version 2.16.0). For flat-mounted retinas, four randomly selected fields were analyzed from the central (<0.9 mm from the optic nerve head) and peripheral (1.7–2.4 mm) regions using QuPath (version 0.5.1). Synaptic colocalization analyses were performed using Imaris 10.2 (Bitplane, Zurich, Switzerland), where the three-dimensional volume of overlapping pre- and postsynaptic markers was quantified using the ImarisColoc module (Bitplane).

### 2.8. Western Blotting

Western blotting was performed using standard Tris-glycine SDS-PAGE protocols. Protein concentrations were determined using a bicinchoninic acid (BCA) assay (Takara, Kusatsu, Shiga, Japan). Retinal lysates were separated by SDS-PAGE and transferred onto PVDF membranes. Membranes were blocked for 20 min at room temperature using a commercial blocking solution (Epizyme, Shanghai, China) and incubated overnight at 4 °C with primary antibodies diluted in antibody diluent (Epizyme). After incubation with horseradish peroxidase-conjugated secondary antibodies (Jackson ImmunoResearch, West Grove, PA, USA), signals were visualized using the Omni-ECL Femto Chemiluminescence Kit (Epizyme) and a ChemiDoc MP Imaging System (Tenon, Shanghai, China). Primary antibodies used included GS (mouse, 1:500, MAB302, MilliporeSigma, Burlington, MA, USA), GLAST (rat, 1:2000, OB-PRT025, Oasis, Taizhou, Zhejiang, China), and α-tubulin (mouse, 1:2000, S0B0307, Starter, Hangzhou, Zhejiang, China).

### 2.9. GS Activity Assay

Retinas were harvested, washed with PBS, weighed, and homogenized in extraction buffer at a ratio of 10 mL per gram of tissue. Homogenates were centrifuged at 13,000× *g* rpm for 30 min, and supernatants were collected for enzymatic analysis. GS activity was measured using a colorimetric assay according to the manufacturer’s instructions (GS Activity Assay Kit, BC0910; Solarbio, Beijing, China).

### 2.10. RNA-Sequencing

Total RNA was isolated from individual retinas using TRIzol reagent (Invitrogen, Waltham, MA, USA). RNA purity and concentration were assessed with a NanoDrop 2000 spectrophotometer (Thermo Fisher, Waltham, MA, USA), and RNA integrity was evaluated using an Agilent 2100 Bioanalyzer (Agilent, Santa Clara, CA, USA). Libraries were prepared using the TruSeq Stranded mRNA LT Sample Prep Kit (Illumina, San Diego, CA, USA) following the manufacturer’s instructions. RNA sequencing and bioinformatic analyses were performed by OE Biotech Co., Ltd. (Shanghai, China).

### 2.11. Real-Time Quantitative PCR

Total retinal RNA was extracted using TRIzol reagent and reverse-transcribed into cDNA using the PrimeScript RT Reagent Kit (Takara, Kusatsu, Shiga, Japan). RT-qPCR was performed using the TB Green system (Takara). Relative gene expression levels were calculated using the comparative Ct method and normalized to HPRT expression. Primer sequences are listed in Table S1.

### 2.12. Statistical Analysis

Image analysis was performed using ImageJ, and statistical analyses were conducted with GraphPad Prism 10 (Boston, MA, USA). Data are presented as mean ± SEM from at least three independent experiments. Statistical comparisons were performed using unpaired two-tailed Student’s *t*-tests or two-way ANOVA followed by Bonferroni’s multiple-comparison tests, as appropriate. Detailed statistical parameters are provided in the corresponding figure legends. Statistical significance was defined as ns, not significant; * *p* < 0.05; ** *p* < 0.01; *** *p* < 0.001; **** *p* < 0.0001.

## 3. Results

### 3.1. Early Retinal Dysfunction Occurs in the Absence of Local Amyloid Plaque Deposition Along the Visual Pathway in APP/PS1 Mice

APP/PS1 mice exhibit early-onset cerebral amyloidosis, with robust β-amyloid (Aβ) plaque deposition in multiple brain regions, as previously reported [23,24,25]. To determine whether comparable pathology is present in the retina and visual pathway, we performed immunostaining for Aβ using 6E10 and Aβ_1–42_ antibodies in young and aged mice. As expected, age-dependent amyloid plaque accumulation was readily detected in visual-related brain regions, including the visual cortex, lateral geniculate nucleus, pretectum, and superior colliculus. Quantitative analysis revealed that among these regions, overt amyloid plaques were detectable only in the visual cortex of young APP/PS1 mice, whereas significant Aβ accumulation was observed in all four regions in aged APP/PS1 mice (Figure S1A). In contrast, no plaque-like Aβ deposits were detected in the retina of either young or aged APP/PS1 mice (Figure S1B). We further examined Aβ burden in the optic nerve, which anatomically connects the retina and brain. Sparse Aβ-positive signals were observed in the optic nerve of aged APP/PS1 mice; however, these signals lacked plaque-like morphology (Figure S1B). Taken together, these results indicate that along the visual pathway, amyloid plaques first emerge in the visual cortex and remain absent from the retina, while the optic nerve may serve as a potential conduit for Aβ transport from the brain to the eye.

**Figure 1:**
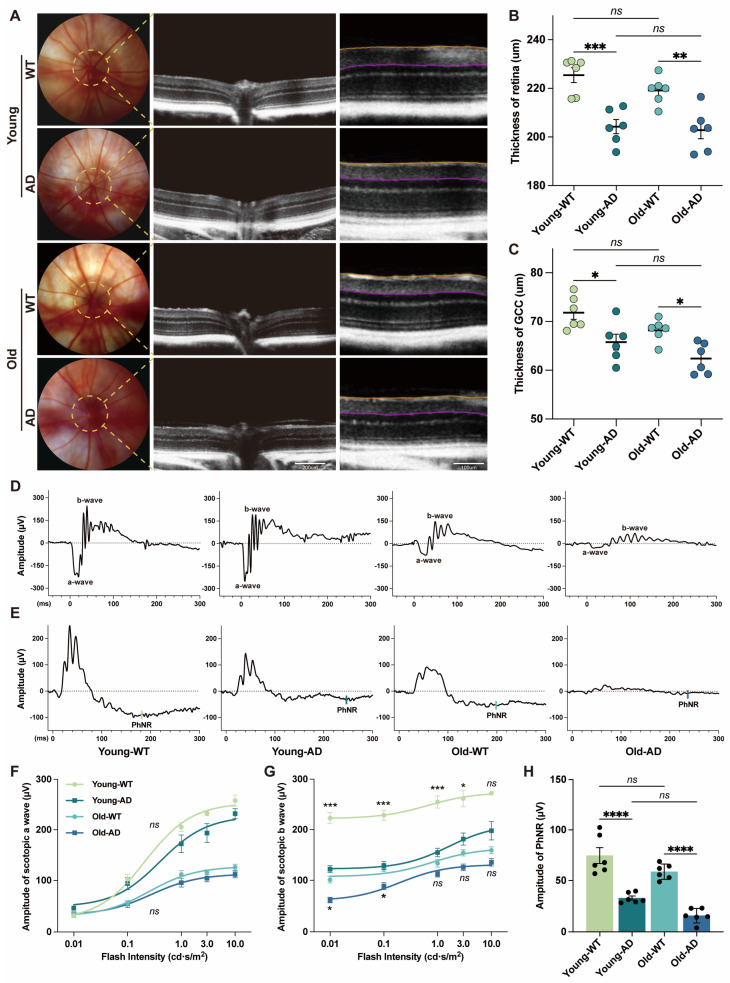
**Structural and functional alterations in the retinas of APP/PS1 mice**. (**A**) Representative fundus photographs and optical coherence tomography (OCT) images from WT and APP/PS1 mice. Colored lines denote automated segmentation used to quantify ganglion cell complex (GCC) thickness. Scale bars: 200 µm (middle) and 100 µm (right). (**B**,**C**) Quantification of total retinal thickness and GCC thickness in WT and APP/PS1 mice at young and old ages (n = 6 mice per group). (**D**) Representative scotopic electroretinogram (ERG) traces recorded at a flash intensity of 3.0 cd·s/m^2^. (**E**) Representative photopic negative response (PhNR) traces recorded at a flash intensity of 10.0 cd·s/m^2^. (**F**,**G**) Quantitative comparison of scotopic a-wave and b-wave amplitudes between WT and APP/PS1 mice at young and old ages (n = 6 mice per group). (**H**) Quantification of PhNR amplitudes (n = 6 mice per group). Data are presented as mean ± SEM. ns, not significant; * *p* < 0.05; ** *p* < 0.01; *** *p* < 0.001; **** *p* < 0.0001. Statistical significance was determined by two-way ANOVA followed by Bonferroni’s multiple-comparison test.

Despite the absence of local amyloid plaque deposition, we observed marked structural and functional impairments in the retina of APP/PS1 mice. Retinal morphology was assessed using OCT and fundus imaging (Figure 1A). OCT volume scans revealed a significant reduction in total retinal thickness in both young and aged APP/PS1 mice compared with age-matched controls (Figure 1B). Thickness of the ganglion cell complex (GCC) was likewise significantly decreased at both ages (Figure 1C), indicating early structural alterations predominantly affecting inner retinal layers.

To evaluate retinal function, ERG and PhNR recordings were performed (Figure 1D,E). Under scotopic conditions, a-wave amplitudes were unchanged, suggesting preserved photoreceptor function (Figure 1F). In contrast, b-wave amplitudes were significantly reduced in young APP/PS1 mice across stimulus intensities of 0.01–3.0 cd·s/m^2^, while aged APP/PS1 mice exhibited reductions at lower luminance levels (0.01 and 0.1 cd·s/m^2^; Figure 1G). At higher luminance (10 cd·s/m^2^), b-wave amplitudes were comparable between groups. Under photopic conditions (10 cd·s/m^2^), a-wave amplitudes were significantly reduced in both young and aged APP/PS1 mice, indicating impaired cone-driven photoreceptor responses (Figure S1E). Photopic b-wave amplitudes were also reduced in young APP/PS1 mice; however, in aged animals, the genotype difference was no longer significant, suggesting a predominant effect of age-related decline (Figure S1F). In addition, PhNR amplitudes recorded at 10 cd·s/m^2^ were significantly reduced in both young and aged APP/PS1 mice relative to controls, indicating impaired RGC-associated function.

Collectively, these findings demonstrate that retinal structural and functional deficits emerge at early stages of AD pathology, despite the absence of detectable amyloid plaque deposition in the retina.

### 3.2. Reduced Retinal Ganglion Cell Density and Impaired Excitatory Synaptic Inputs in APP/PS1 Mice

**Figure 2:**
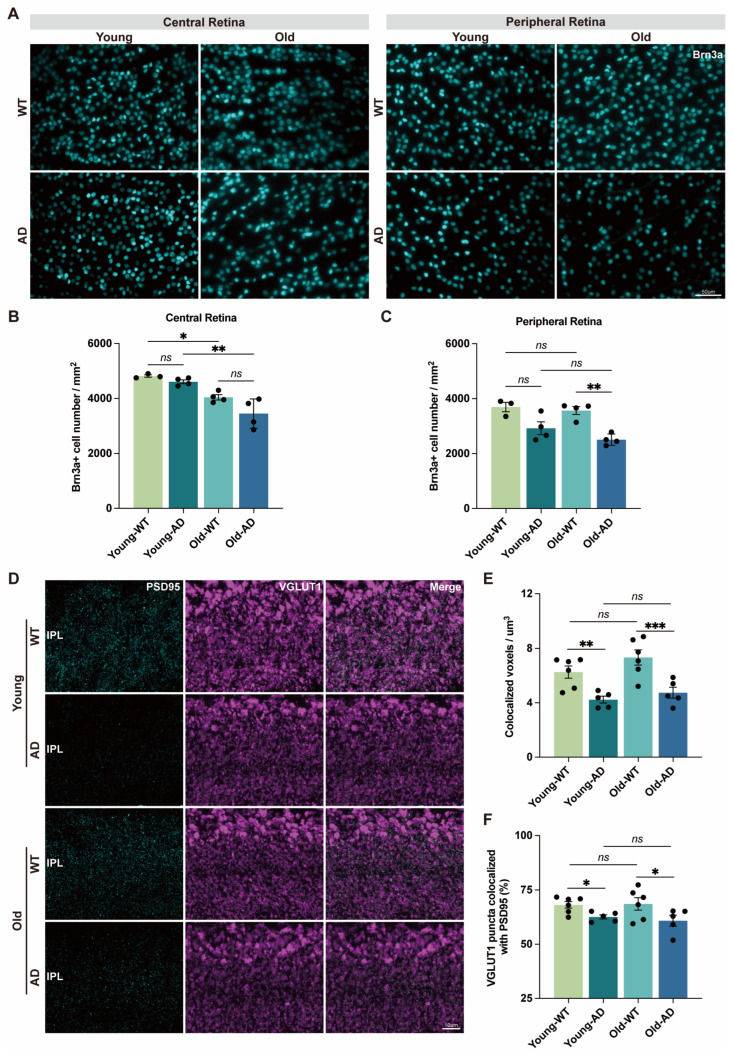
**Reduced retinal ganglion cell density and impaired excitatory synaptic inputs in APP/PS1 mice**. (**A**) Representative retinal whole-mount images showing Brn3a-immunopositive retinal ganglion cells (RGCs) in central and peripheral regions of WT and APP/PS1 retinas. Scale bar: 50 µm. (**B**,**C**) Quantification of Brn3a+ RGC density in central and peripheral retinal regions at young and old ages (n = 3–4 mice per group). (**D**) Representative immunofluorescence images of postsynaptic density protein 95 (PSD95) and vesicular glutamate transporter 1 (VGLUT1) in the inner plexiform layer (IPL). Merged images indicate colocalized pre- and postsynaptic puncta. Scale bar: 10 µm. (**E**,**F**) Quantitative analysis of excitatory synaptic contacts between bipolar cells and RGCs in the IPL. (**E**) Volume of colocalized PSD95+/VGLUT1+ voxels per µm^3^ (n = 5–6 mice per group). (**F**) Proportion of VGLUT1 puncta located within 0.2 µm of PSD95 puncta (n = 5–6 mice per group). Data are presented as mean ± SEM. ns, not significant; * *p* < 0.05; ** *p* < 0.01; *** *p* < 0.001. Statistical significance was determined by two-way ANOVA followed by Bonferroni’s multiple-comparison test.

RGCs constitute the sole output neurons of the retina, transmitting visual information to the brain via their axons in the optic nerve [27]. Given the functional decline of RGCs observed in APP/PS1 mice, we next examined RGC survival in retinal whole mounts, focusing on both central and peripheral regions. Quantification of Brn3a-positive neurons revealed that RGC density in the central retina did not differ significantly between APP/PS1 and WT mice at either young or old ages. In contrast, RGC density in peripheral retinal regions was significantly reduced in aged APP/PS1 mice compared with age-matched WT controls (Figure 2A–C). These results suggest that RGC loss exhibits regional selectivity, with peripheral RGCs being more susceptible to AD-associated pathology, whereas central RGC density appears to be predominantly influenced by aging.

Previous studies have shown that a reduction in glutamatergic synapse density represents an early event in RGC degeneration, preceding dendritic retraction, axonal pathology, and somatic loss [14,15]. To determine whether synaptic alterations occur at early stages of AD-associated retinal pathology, we assessed excitatory synaptic inputs to RGCs by immunolabeling postsynaptic density protein 95 (PSD95), which is enriched in RGC dendrites, and vesicular glutamate transporter 1 (VGLUT1), a presynaptic marker of bipolar cell ribbon synapses [28]. PSD95 immunoreactivity within the inner plexiform layer was markedly reduced in both young and aged APP/PS1 retinas compared with WT controls (Figure 2D). 

To quantify synaptic contacts, we measured the colocalized volume of PSD95 and VGLUT1 puncta and calculated the fraction of VGLUT1 puncta located within a physiologically relevant distance (0.2 μm) of PSD95 puncta in the inner plexiform layer. Both measures were significantly reduced in young and aged APP/PS1 retinas (Figure 2E,F), indicating a loss of excitatory synaptic connectivity. Together, these findings demonstrate that impaired excitatory synaptic inputs to RGCs emerge early during AD-associated retinal pathology and precede overt loss of RGC somata.

### 3.3. Altered Expression of Müller Glial Markers in the Retina of APP/PS1 Mice

**Figure 3:**
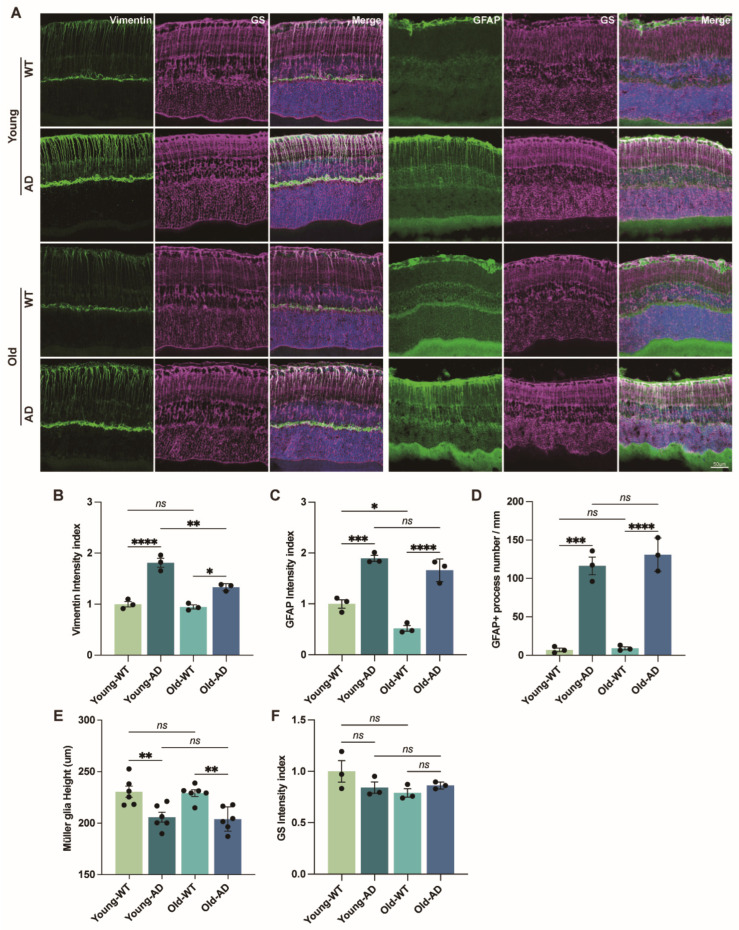
**Reactive changes in Müller glia in the retinas of APP/PS1 mice**. (**A**) Representative confocal images of retinal sections immunolabeled for vimentin and glial fibrillary acidic protein (GFAP), together with the Müller glial marker glutamine synthetase (GS). Scale bar: 50 µm. (**B**,**C**) Quantification of vimentin and GFAP immunofluorescence intensity in WT and APP/PS1 retinas (n = 3 mice per group). (**D**) Quantification of GFAP-positive Müller glial processes traversing the inner plexiform layer (IPL), expressed as the number of processes per millimeter of retinal length (n = 3 mice per group). (**E**) Measurement of Müller glial radial extension, defined as the distance from the inner limiting membrane (ILM) to the external limiting membrane (ELM) (n = 6 mice per group). (**F**) Quantification of GS immunofluorescence intensity in WT and APP/PS1 retinas (n = 3 mice per group). Data are presented as mean ± SEM. ns, not significant; * *p* < 0.05; ** *p* < 0.01; *** *p* < 0.001; **** *p* < 0.0001. Statistical significance was determined by two-way ANOVA followed by Bonferroni’s multiple-comparison test.

Müller glia span the entire thickness of the neural retina and have been proposed to act as early responders to AD-related retinal pathology, potentially contributing to RGC degeneration [17,18,29]. To characterize Müller glial activation in APP/PS1 mice, we examined the expression of GFAP and vimentin in WT and AD retinas. In the healthy retina, Müller glia express minimal levels of GFAP, whereas both GFAP and vimentin are upregulated in activated Müller glia following retinal injury or stress [30,31]. In APP/PS1 mice, vimentin immunoreactivity was significantly increased in both young and aged retinas and was broadly distributed across the GCL and IPL. Vimentin immunoreactivity was markedly increased in AD retinas and displayed a prominent filamentous distribution along Müller glial processes, colocalizing with the Müller glial marker GS (Figure 3A,B).

Consistent with this, double immunolabeling for GS and GFAP revealed prominent GFAP localization along Müller glial processes within the IPL, accompanied by marked upregulation of GFAP in both young and aged APP/PS1 retinas (Figure 3A,C). Following established methods [32], we quantified the number of GFAP-positive Müller glial processes traversing the IPL and observed a significant increase in both young and aged APP/PS1 mice compared with WT controls (Figure 3D).

Whole-mount GFAP immunostaining further demonstrated pronounced activation of Müller glial endfeet in young APP/PS1 mice. Notably, the density of GFAP-positive endfeet was higher in peripheral retinal regions than in central regions (Figure S2A), indicating regional heterogeneity in Müller glial activation. Together, these findings confirm robust Müller glial activation in the retina under AD-associated pathology.

We next asked whether this reactive Müller glial response was accompanied by cell proliferation. Retinal sections were co-immunostained for Ki67, a marker of cell proliferation, and GS. Across all genotypes and ages examined, no Ki67-positive GS-expressing Müller glia were detected (Figure S2B), indicating that Müller glial activation in APP/PS1 retinas occurs without a proliferative response.

### 3.4. Morphological Remodeling of Reactive Müller Glia in the Retina of Young APP/PS1 Mice

Cell morphology is closely linked to cellular function, particularly for glial cells, whose complex architecture underlies their ability to contact, support, and regulate neuronal networks. Because GS immunolabeling reliably outlines the full extent of Müller glial processes, we first quantified Müller glial height, defined as the distance between the inner limiting membrane (ILM) and the external limiting membrane (ELM). Müller glial height was significantly reduced in both young and aged APP/PS1 mice compared with age-matched WT controls (Figure 3E), indicating global morphological alterations associated with AD pathology.

**Figure 4:**
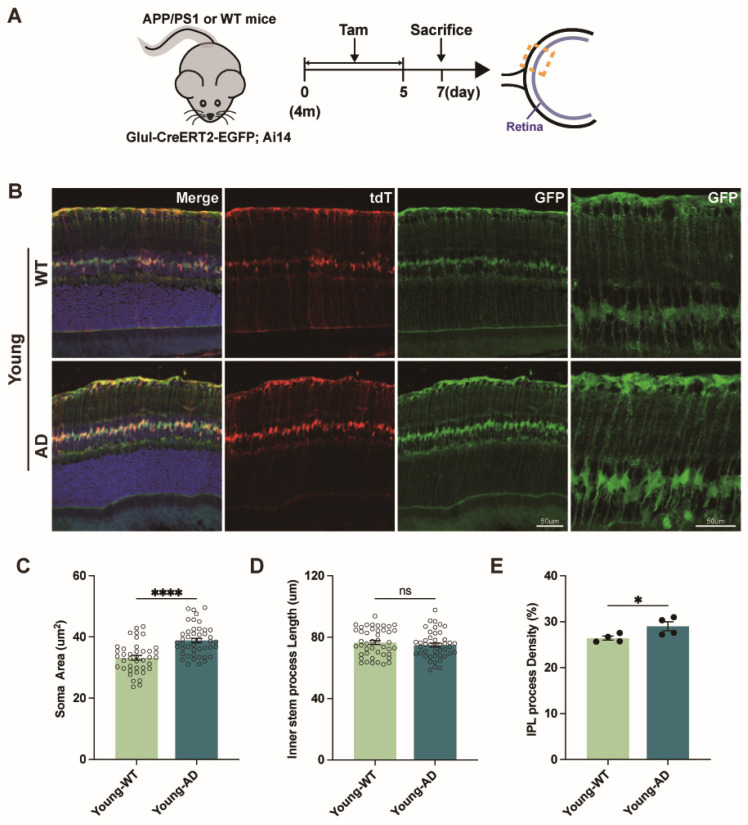
**Genetic tracing reveals morphological remodeling of reactive Müller glia in the retinas of young APP/PS1 mice**. (**A**) Schematic illustrating the generation of *Glul*-CreERT2-EGFP; Ai14; APP/PS1 mice and the experimental timeline for tamoxifen (Tam)-induced genetic tracing and morphological analysis of tdTomato-positive Müller glia in WT and APP/PS1 retinas. (**B**) Representative confocal images of genetically labeled Müller glia. tdTomato labels Müller glial somata and primary stem processes, whereas EGFP highlights lateral side processes. Scale bar: 50 µm. (**C**) Quantification of Müller glial soma area within the inner nuclear layer (INL) (n = 40–50 cells from 12 retinal sections from 4 mice per group). (**D**) Quantification of inner stem process length, measured from the soma to the ganglion cell layer (GCL) (n = 40–50 cells from 12 retinal sections from 4 mice per group). (**E**) Quantification of Müller glial process density in the inner plexiform layer (IPL), expressed as the percentage of IPL area occupied by EGFP-positive processes (n = 4 mice per group). Data are presented as mean ± SEM. ns, not significant; * *p* < 0.05; **** *p* < 0.0001. Statistical significance was assessed using an unpaired two-tailed Student’s *t*-test.

To exclude potential artifacts related to antibody-based labeling, we next employed a genetic tracing strategy. We generated Glul-CreERT2-EGFP; Ai14 mice and crossed them with APP/PS1 mice to obtain Glul-CreERT2-EGFP; Ai14; APP/PS1 mice, enabling tamoxifen-inducible genetic labeling of Müller glia. Four-month-old Glul-CreERT2-EGFP; Ai14; APP/PS1 and WT littermates received tamoxifen for five consecutive days and were sacrificed two days after the final injection (Figure 4A).

In control retinas, Müller glia exhibited their characteristic radial morphology, with somata located in INL, an inner stem process extending toward GCL, and an outer stem process projecting toward ONL. From these stem processes, Müller glia elaborate numerous specialized side processes that enable extensive interactions with retinal neurons [16]. Because our analyses focused on alterations within the inner retina, we quantified Müller glial soma area, the length of the inner stem process, and the density of both stem and side processes within the IPL.

Quantitative analysis revealed a significant increase in Müller glial soma size and increased process density within the IPL in young APP/PS1 mice compared with WT controls (Figure 4B,C,E). Although overall Müller glial height was reduced, the length of the inner stem process was not significantly altered (Figure 4B,D). Together, these findings indicate early morphological remodeling of Müller glia in APP/PS1 mice, characterized by somatic hypertrophy and increased inner retinal process complexity.

### 3.5. Increased GS Activity and Disrupted AQP4 Polarity in the Retina of APP/PS1 Mice

**Figure 5:**
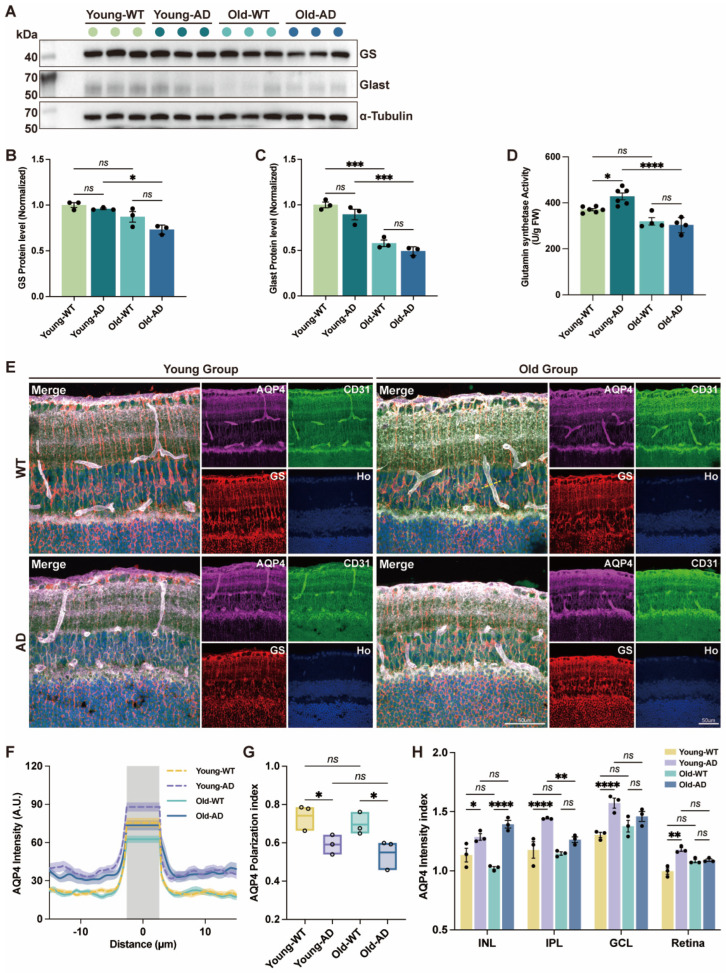
**Increased GS activity and disrupted AQP4 polarity in AD retinas**. (**A**) Representative immunoblots showing GS and GLAST expression in retinas from WT and APP/PS1 mice at young and old ages. (**B**,**C**) Densitometric quantification of GS (**B**) and GLAST (**C**) protein levels (n = 3 mice per group). Band intensities were normalized to α-tubulin and expressed relative to young WT controls. (**D**) GS enzymatic activity measured in freshly isolated retinal tissue from WT and APP/PS1 mice at young and old ages (n = 4–6 mice per group). (**E**) Representative confocal images of retinal sections immunolabeled for AQP4, the endothelial marker CD31, and GS. (**F**) Line-scan analysis of AQP4 fluorescence intensity along retinal blood vessels (yellow dashed lines in (**E**)). (**G**) Quantification of AQP4 polarization index, defined as the difference between peak perivascular fluorescence and baseline parenchymal fluorescence, normalized to the peak value (n = 3 mice per group). (**H**) Quantification of AQP4 immunofluorescence intensity in the inner nuclear layer (INL), inner plexiform layer (IPL), ganglion cell layer (GCL), and across the entire neural retina (n = 3 mice per group). Data are presented as mean ± SEM. ns, not significant; * *p* < 0.05; ** *p* < 0.01; *** *p* < 0.001; **** *p* < 0.0001. Statistical significance was determined by two-way ANOVA followed by Bonferroni’s multiple-comparison test.

Müller glia provide essential structural and metabolic support for retinal neurons and play a central role in limiting excitotoxicity and maintaining ion and fluid homeostasis [16]. Given the pronounced morphological remodeling observed in the AD retina, we next asked whether key Müller glial functions were correspondingly altered. Efficient glutamate recycling is critical for normal retinal function and neuroprotection. Müller glia express high levels of the glutamate/aspartate transporter GLAST, which mediates glutamate uptake, and GS, which converts intracellular glutamate to glutamine [18]. Immunofluorescence analysis revealed no significant difference in GS immunoreactivity between APP/PS1 and WT retinas (Figure 3F). Consistently, Western blot analysis showed that GS and GLAST protein levels were comparable between APP/PS1 and age-matched WT retinas. However, both GS and GLAST protein expression were significantly reduced in aged APP/PS1 retinas compared with young APP/PS1 retinas (Figure 5A–C), indicating an age-dependent decline.

We next directly assessed GS enzymatic function using freshly isolated retinal tissue. GS activity was significantly increased in young APP/PS1 retinas relative to WT controls, whereas GS activity in aged APP/PS1 mice did not differ from that of age-matched WT mice (Figure 5D). These findings indicate that GS enzymatic activity is selectively enhanced at early stages of AD-associated retinal pathology, despite unchanged protein abundance.

In addition to neurotransmitter metabolism, Müller glia are key regulators of retinal ion and water homeostasis. We therefore examined aquaporin-4 (AQP4), a major glial water channel whose transport function depends on both expression level and polarized subcellular localization [16]. Because AQP4 is enriched in Müller glial perivascular processes and basal endfeet, we performed triple immunofluorescent labeling for GS, CD31, and AQP4 to assess its spatial distribution relative to retinal vasculature. Quantitative analysis revealed increased AQP4 expression in the INL, IPL, GCL, and across the entire neural retina in young APP/PS1 mice. In aged APP/PS1 mice, AQP4 expression remained elevated in the INL but did not differ significantly from WT controls in the IPL, GCL or whole retina (Figure 5E,H). To evaluate AQP4 polarity, we calculated a polarization index as previously described, defined as the difference between peak perivascular fluorescence and baseline fluorescence, normalized to the peak value [22]. Although both perivascular and non-vascular AQP4 signals were increased in APP/PS1 retinas, the polarization index was significantly reduced in both young and aged APP/PS1 mice compared with WT controls (Figure 5E–G), indicating a disruption of AQP4 polarity.

Together, these results demonstrate that AD-associated retinal pathology is accompanied by early alterations in Müller glial metabolic activity and a sustained loss of AQP4 polarization, changes that are likely to compromise retinal water homeostasis and glymphatic transport efficiency.

### 3.6. Transcriptomic Profiling Reveals Molecular Alterations in the Retinas of Young APP/PS1 Mice

**Figure 6:**
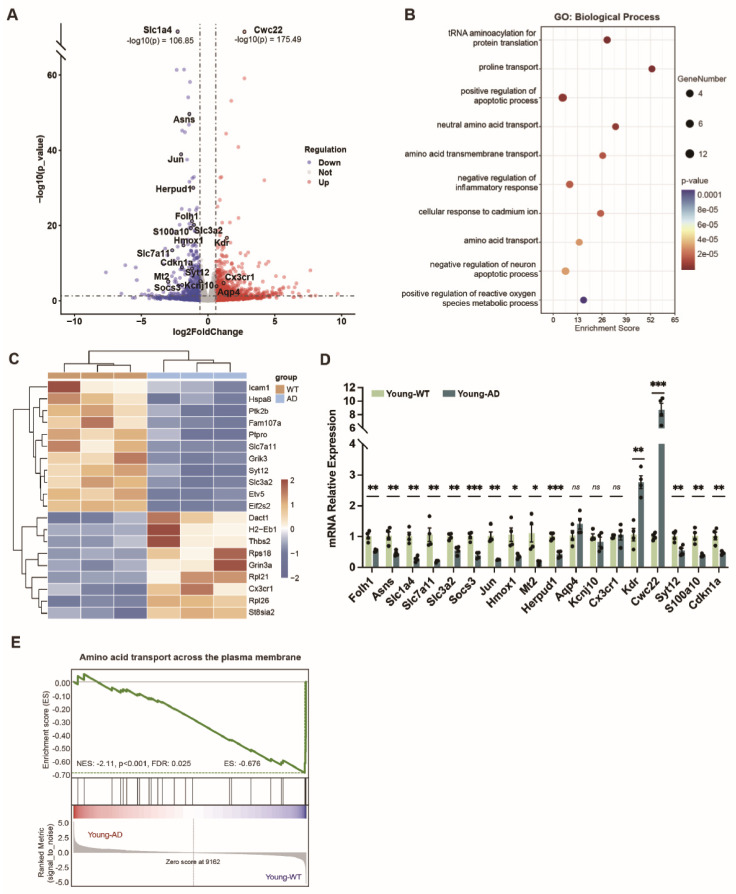
**Transcriptomic alterations in the retinas of young APP/PS1 mice**. (**A**) Volcano plot showing differentially expressed genes (DEGs) in retinas from young APP/PS1 mice relative to age-matched WT controls. (**B**) Gene Ontology (GO) enrichment analysis of DEGs, highlighting pathways associated with amino acid transport, apoptotic regulation, inflammatory responses, and oxidative stress. (**C**) Heatmap showing synapse-associated DEGs derived from GO enrichment (n = 3 per group). (**D**) RT-qPCR validation of selected DEGs involved in amino acid metabolism and transport, neuronal apoptosis, and oxidative stress (n = 4 per group). Data are presented as mean ± SEM. ns, not significant; * *p* < 0.05; ** *p* < 0.01; *** *p* < 0.001; unpaired two-tailed *t*-test. (**E**) Gene Set Enrichment Analysis (GSEA) of the retinal transcriptome showing significant negative enrichment of the Reactome pathway “Amino acid transport across the plasma membrane” in young APP/PS1 retinas compared with WT controls.

To investigate molecular pathways associated with early AD-related retinal pathology, we performed RNA sequencing (RNA-seq) on retinal tissue from young APP/PS1 and age-matched WT mice. Differential expression analysis identified 376 upregulated and 228 downregulated genes in the retinas of young APP/PS1 mice relative to WT controls (Figure 6A and Figure S3A,B).

Gene ontology (GO) enrichment analysis of differentially expressed genes highlighted pathways related to amino acid transport, regulation of apoptotic processes, inflammatory responses, and reactive oxygen species metabolism (Figure 6B), in agreement with the functional pathways enriched in the KEGG analysis (Figure S3C). In light of the early synaptic vulnerability of retinal ganglion cells indicated by our phenotypic analyses, we further examined synapse-related GO categories and their leading-edge genes, identifying enrichment of multiple terms associated with synapse organization and assembly (Figure 6C). To validate the transcriptomic findings, RT-qPCR was performed on a subset of differentially expressed genes involved in amino acid transport and metabolism, neuronal apoptosis, and oxidative stress, yielding results consistent with the RNA-seq data (Figure 6D).

To further assess coordinated pathway-level changes, we conducted Gene Set Enrichment Analysis (GSEA). This analysis revealed significant negative enrichment of the Reactome pathway “Amino acid transport across the plasma membrane” in young APP/PS1 retinas compared with WT controls (normalized enrichment score [NES] = −2.11, *p* < 0.001, false discovery rate [FDR] = 0.025), indicating a global downregulation of genes involved in amino acid transport (Figure 6E).

## 4. Discussion

In the present study, we demonstrate that retinal structural, synaptic, and glial abnormalities emerge early in APP/PS1 mice despite the absence of detectable Aβ plaque deposition in the retina. Using multiple complementary approaches, we identify Müller glial activation and remodeling as prominent early features of AD-associated retinal pathology and show that these changes coincide with excitatory synaptic deficits in RGCs that precede neuronal loss.

Consistent with our findings, neither 6E10- nor Aβ_1–42_-positive plaques were detected in the retinas of APP/PS1 mice even at advanced ages. Parallel staining of retinal and brain tissues confirmed robust, age-dependent amyloid plaque deposition in visual-related brain regions, particularly in the visual cortex, supporting the sensitivity of our detection methods. Nevertheless, the absence of plaque-like Aβ aggregates does not exclude the presence of non-fibrillar Aβ species in the retina. Previous studies have reported soluble Aβ oligomers in the retinas of young APP/PS1 mice in the absence of retinal plaques, whereas plaques emerge later and are primarily observed in the brain [33]. Similarly, in AppNLGF mice, soluble retinal Aβ oligomers are detectable at early stages, whereas plaque deposition only appears after 18 months of age [34]. Given the limitations of our immunohistochemical approaches, we cannot directly link Müller glial alterations to specific Aβ species or distributions in the retina.

The markedly lower Aβ burden in the retina compared with the brain may reflect fundamental differences in local APP processing. APP and presenilin 1 (PS1), a core component of the γ-secretase complex, are upregulated in the brains of AD patients and mouse models but show no significant changes in expression in the retina [22]. In addition, Aβ has been proposed to reach the eye from the brain via the optic nerve, perivascular spaces, and optic nerve sheath [22]. In line with this model, we detected sparse Aβ-positive signals in the optic nerve of aged APP/PS1 mice, supporting the idea that retinal exposure to Aβ may occur indirectly rather than through local plaque formation.

Despite the absence of retinal plaques, young APP/PS1 mice already exhibited thinning of inner retinal layers, impaired retinal function, and reduced excitatory synaptic inputs to RGCs. Synaptic dysfunction is increasingly recognized as an early and critical event in neurodegenerative diseases, often preceding neuronal loss. In glaucoma, synapse disassembly represents one of the earliest pathological changes in RGC degeneration [14,15]. Our findings extend this concept to AD-associated retinal pathology and suggest that synaptic vulnerability of RGCs may represent an early and sensitive indicator of retinal neurodegeneration.

Although the present study primarily focused on RGC injury and Müller glial reactivity, our ERG data also provide insight into photoreceptor-related dysfunction in APP/PS1 retinas. Together, the scotopic and photopic ERG findings suggest a pattern of multi-level retinal dysfunction, characterized by rod-pathway postreceptoral abnormalities together with cone-pathway photoreceptor impairment. Specifically, the relatively preserved scotopic a-wave, in contrast to the reduced photopic a-wave, suggests that cone-driven photoreceptor responses are more vulnerable in APP/PS1 mice. This observation is noteworthy because cones rely not only on the canonical RPE visual cycle but also on a retina-based pathway involving Müller cell-associated retinoid processing to sustain rapid pigment regeneration under daylight conditions [35,36,37]. Therefore, when considered together with the Müller glial molecular alterations observed in our study, the photopic deficit is consistent with impaired glial support for cone function, potentially including disruption of cone visual pigment recycling [38]. However, as retinoid flux, dark adaptation kinetics, and specific visual-cycle components were not directly assessed, these findings should be interpreted as suggesting a plausible mechanistic link between Müller cell dysfunction and altered cone visual cycling, rather than as direct proof of this mechanism. 

By contrast, the age-related decline in scotopic a-wave amplitude is more likely to reflect aging-associated rod dysfunction. Previous studies have shown that in WT C57BL/6 mice, rod photoreceptor function declines with aging and rod number also decreases, whereas no obvious age-dependent change in cone number has been observed [39]. This may help explain why rod-dominant functional decline can occur even when the overall outer retinal structure appears relatively preserved. Consistent with this interpretation, previous work has reported no significant age-dependent change in whole-retinal thickness by OCT in WT mice [40]. Thus, our data suggest that AD-related pathology may preferentially compromise cone-associated function, potentially in association with Müller cell dysfunction, whereas rod-associated impairment appears to be more strongly influenced by aging.

A key finding of this study is the early and regionally heterogeneous activation of Müller glia. Müller glial gliosis has been implicated in RGC degeneration across multiple retinal diseases, including glaucoma, diabetic retinopathy, and multiple sclerosis [18,41,42]. In young APP/PS1 mice, we observed a higher density of GFAP-positive Müller glial endfeet in the peripheral retina compared with the central retina. Notably, this spatial pattern mirrored the regional loss of RGC somata observed at later stages, with peripheral RGCs showing greater vulnerability. This correspondence supports an association between Müller glial activation and RGC degeneration in the AD retina, although causal relationships remain to be established.

Beyond marker expression, we provide direct evidence that reactive Müller glia undergo substantial morphological remodeling in the AD retina. Glial morphology is tightly linked to function, as the elaborate architecture of Müller glia enables extensive contact with neuronal synapses, blood vessels, and extracellular space. Using an endogenous genetic labeling strategy, we quantified Müller glial soma size, stem process organization, and process density in the inner plexiform layer. Müller glia in APP/PS1 retinas displayed soma hypertrophy and increased process density. These structural changes are likely to alter glia–neuron and glia–vasculature interactions and may influence synaptic stability and metabolic support. However, our morphological analysis was limited to young adult mice. Future studies in aged APP/PS1 retinas will be necessary to determine whether these remodeling changes progress further with aging.

We next examined functional consequences of Müller glial activation, focusing on glutamate metabolism and fluid homeostasis. GS plays a critical role in glutamate–glutamine cycling and neuroprotection in the retina [16,43]. Although GS protein levels were unchanged between age-matched AD and WT retinas, GS enzymatic activity was selectively increased in young APP/PS1 mice and declined with disease progression. These findings contrast with reports of reduced GS activity in postmortem AD retinas [20] and may reflect stage-dependent differences between early and late pathology. One possible explanation is that elevated GS activity in young AD retinas represents a compensatory response to increased synaptic stress or altered glutamate handling. Whether this response is protective or ultimately insufficient to prevent excitotoxic injury remains to be determined.

In parallel, we identified altered expression and disrupted polarity of AQP4. Polarization of AQP4 at perivascular Müller glial endfeet is essential for retinal fluid balance and glymphatic transport [44]. Although AQP4 expression was increased in AD retinas, its perivascular polarization was significantly reduced, suggesting impaired spatial organization rather than simple upregulation. Transcriptomic analysis further revealed downregulation of Kcnj10, encoding the inwardly rectifying potassium channel Kir4.1, which functionally cooperates with AQP4. While our study did not directly assess Kir4.1 function, these findings point to broader disruptions of Müller glial ion and water homeostasis in AD pathology.

Among the downregulated DEGs, Slc7a11 and Slc3a2, which encode the two subunits of the cystine/glutamate antiporter system Xc−, were prominently reduced in the heat map analysis (Figure 6A). Given the essential role of system Xc− in maintaining intracellular glutathione levels and antioxidant defense, their downregulation suggests impaired redox homeostasis and a potential increased vulnerability to ferroptosis in APP/PS1 retinas [45]. Consistently, KEGG pathway analysis of downregulated genes revealed significant enrichment of the ferroptosis pathway (Figure S3C). In addition, Hmox1, encoding heme oxygenase and involved in heme degradation and iron metabolism, may reflect altered iron homeostasis and oxidative stress responses [46]. We also observed altered expressions of Mt1 and Mt2, which are associated with antioxidant defense and have been implicated in protection against oxidative stress-related injury [47]. Collectively, these changes suggest that impaired antioxidant capacity, redox imbalance, and ferroptosis-related mechanisms may contribute to retinal pathology in APP/PS1 mice.

In summary, our study demonstrates that APP/PS1 mice develop early retinal dysfunction characterized by synaptic deficits, Müller glial activation, and metabolic and homeostatic alterations despite the absence of retinal Aβ plaque deposition. By combining structural, functional, molecular, and genetic approaches, we provide a comprehensive characterization of Müller glial remodeling in the AD retina. These findings suggest that Müller glia may contribute to neuronal vulnerability and disease progression, and highlight retinal glia as potential targets for early diagnosis and therapeutic intervention in Alzheimer’s disease.

## Data Availability

The original contributions presented in this study are included in the article/Supplementary Materials. Further inquiries can be directed to the corresponding authors.

## Supplementary Materials

The following supporting information can be downloaded at: https://www.mdpi.com/article/10.3390/cells15090801/s1, Table S1: Primers (PCR, RT-qPCR). Figure S1: Differential amyloid β distribution along the visual pathway in APP/PS1 mice. (A) Representative confocal images showing co-immunostaining for 6E10 and Aβ_1–42_ in the visual cortex (VC), lateral geniculate nucleus (LGN), pretectum (PRT), and superior colliculus (SC) of young and old APP/PS1 mice. Dashed lines indicate anatomical boundaries of each visual-related nucleus. Scale bars: 200 μm (VC, LGN, PRT) and 400 μm (SC). (B) Representative images of 6E10 and Aβ_1–42_ immunostaining in the retina and optic nerve of young and old APP/PS1 mice. Scale bar: 50 μm. (C,D) Quantification of 6E10- and Aβ_1–42_-positive area, expressed as the percentage of total region area, in VC, LGN, PRT, and SC (n = 5–6 mice per group). (E,F) Quantitative comparison of photopic a-wave and b-wave amplitudes between WT and APP/PS1 mice at young and old ages (n = 4 mice per group). Data are presented as mean ± SEM. * *p* < 0.05; *** *p* < 0.001; **** *p* < 0.0001; two-way ANOVA followed by Bonferroni’s multiple-comparison test. Figure S2: Absence of proliferative responses in reactive Müller glia in APP/PS1 retinas. (A) Representative whole-mount images of GFAP-immunopositive macroglia in the central and peripheral retina. Activated Müller glial endfeet are indicated by arrowheads. Scale bar: 50 μm. (B) Representative retinal sections immunolabeled for Ki67 and GS, showing no Ki67-positive Müller glia. Scale bar: 50 μm. Figure S3: Transcriptomic alterations in the retinas of young APP/PS1 mice revealed by RNA sequencing. (A) Number of significantly upregulated and downregulated differentially expressed genes (DEGs) identified in retinal RNA-seq analysis. (B) Heatmap depicting all significant DEGs detected in the retinal transcriptome (n = 3). (C) Kyoto Encyclopedia of Genes and Genomes (KEGG) pathway enrichment analysis of downregulated DEGs, highlighting pathways related to amino acid metabolism, inflammatory signaling, and cellular stress responses.

## Abbreviations

The following abbreviations are used in this manuscript:

Aβ	Amyloid beta
AD	Alzheimer’s disease
APP	Amyloid precursor protein
AQP4	Aquaporin-4
CNS	Central nervous system
ERG	Electroretinography
GCC	Ganglion cell complex
GCL	Ganglion cell layer
GFAP	Glial fibrillary acidic protein
GLAST	Glutamate/aspartate transporter
GS	Glutamine synthetase
INL	Inner nuclear layer
IPL	Inner plexiform layer
LGN	Lateral geniculate nucleus
OCT	Optical coherence tomography
ONL	Outer nuclear layer
PhNR	Photopic negative response
PRT	Pretectum
PS1	Presenilin 1
PSD95	Postsynaptic density protein 95
RGCs	Retinal ganglion cells
RNFL	Retinal nerve fiber layer
SC	Superior colliculus
VC	Visual cortex
VGLUT1	Vesicular glutamate transporter 1
WT	Wild type

## References

[1] Knopman D.S., Amieva H., Petersen R.C., Chételat G., Holtzman D.M., Hyman B.T., Nixon R.A., Jones D.T. (2021). Alzheimer disease. Nat. Rev. Dis. Primers.

[2] Whitson H.E., Cronin-Golomb A., Cruickshanks K.J., Gilmore G.C., Owsley C., Peelle J.E., Recanzone G., Sharma A., Swenor B., Yaffe K. (2018). American Geriatrics Society and National Institute on Aging Bench-to-Bedside Conference: Sensory Impairment and Cognitive Decline in Older Adults. J. Am. Geriatr. Soc..

[3] Albers M.W., Gilmore G.C., Kaye J., Murphy C., Wingfield A., Bennett D.A., Boxer A.L., Buchman A.S., Cruickshanks K.J., Devanand D.P. (2015). At the interface of sensory and motor dysfunctions and Alzheimer’s disease. Alzheimers Dement..

[4] Nordengen K., Kirsebom B.-E., Henjum K., Selnes P., Gísladóttir B., Wettergreen M., Torsetnes S.B., Grøntvedt G.R., Waterloo K.K., Aarsland D. (2019). Glial activation and inflammation along the Alzheimer’s disease continuum. J. Neuroinflamm..

[5] Wilson D.M., Cookson M.R., Van Den Bosch L., Zetterberg H., Holtzman D.M., Dewachter I. (2023). Hallmarks of neurodegenerative diseases. Cell.

[6] Long J.M., Holtzman D.M. (2019). Alzheimer Disease: An Update on Pathobiology and Treatment Strategies. Cell.

[7] Sperling R., Mormino E., Johnson K. (2014). The Evolution of Preclinical Alzheimer’s Disease: Implications for Prevention Trials. Neuron.

[8] Byerly M.S., Blackshaw S. (2009). Vertebrate retina and hypothalamus development. Wiley Interdiscip. Rev. Syst. Biol. Med..

[9] De Groef L., Cordeiro M.F. (2018). Is the Eye an Extension of the Brain in Central Nervous System Disease?. J. Ocul. Pharmacol. Ther..

[10] Marziani E., Pomati S., Ramolfo P., Cigada M., Giani A., Mariani C., Staurenghi G. (2013). Evaluation of Retinal Nerve Fiber Layer and Ganglion Cell Layer Thickness in Alzheimer’s Disease Using Spectral-Domain Optical Coherence Tomography. Investig. Opthalmol. Vis. Sci..

[11] Doustar J., Torbati T., Black K.L., Koronyo Y., Koronyo-Hamaoui M. (2017). Optical Coherence Tomography in Alzheimer’s Disease and Other Neurodegenerative Diseases. Front. Neurol..

[12] Cheung C.Y., Ong Y.T., Hilal S., Ikram M.K., Low S., Ong Y.L., Venketasubramanian N., Yap P., Seow D., Chen C.L. (2015). Retinal Ganglion Cell Analysis Using High-Definition Optical Coherence Tomography in Patients with Mild Cognitive Impairment and Alzheimer’s Disease. J. Alzheimers Dis..

[13] Tams A.L.M., Sanz-Morello B., Westi E.W., Mouhammad Z.A., Andersen J.V., Freude K.K., Vohra R., Hannibal J., Aldana B.I., Kolko M. (2021). Decreased Glucose Metabolism and Glutamine Synthesis in the Retina of a Transgenic Mouse Model of Alzheimer’s Disease. Cell. Mol. Neurobiol..

[14] Ou Y., Jo R.E., Ullian E.M., Wong R.O., Della Santina L. (2016). Selective Vulnerability of Specific Retinal Ganglion Cell Types and Synapses after Transient Ocular Hypertension. J. Neurosci..

[15] Van Hook M.J. (2022). Influences of Glaucoma on the Structure and Function of Synapses in the Visual System. Antioxid. Redox Signal..

[16] Arrigo A., Cremona O., Aragona E., Casoni F., Consalez G., Dogru R.M., Hauck S.M., Antropoli A., Bianco L., Parodi M.B. (2025). Müller cells trophism and pathology as the next therapeutic targets for retinal diseases. Prog. Retin. Eye Res..

[17] Palko S.I., Benoit M.R., Yao A.Y., Mohan R., Yan R. (2024). ER-stress response in retinal Müller glia occurs significantly earlier than amyloid pathology in the Alzheimer’s mouse brain and retina. Glia.

[18] Miao Y., Zhao G.-L., Cheng S., Wang Z., Yang X.-L. (2023). Activation of retinal glial cells contributes to the degeneration of ganglion cells in experimental glaucoma. Prog. Retin. Eye Res..

[19] Zhang M., Zhong L., Han X., Xiong G., Xu D., Zhang S., Cheng H., Chiu K., Xu Y. (2021). Brain and Retinal Abnormalities in the 5xFAD Mouse Model of Alzheimer’s Disease at Early Stages. Front. Neurosci..

[20] Xu Q.A., Boerkoel P., Hirsch-Reinshagen V., Mackenzie I.R., Hsiung G.R., Charm G., To E.F., Liu A.Q., Schwab K., Jiang K. (2022). Müller cell degeneration and microglial dysfunction in the Alzheimer’s retina. Acta Neuropathol. Commun..

[21] Riepe R.E., Norenburg M.D. (1977). Müller cell localisation of glutamine synthetase in rat retina. Nature.

[22] Cao Q., Yang S., Wang X., Sun H., Chen W., Wang Y., Gao J., Wu Y., Yang Q., Chen X. (2024). Transport of β-amyloid from brain to eye causes retinal degeneration in Alzheimer’s disease. J. Exp. Med..

[23] Li K., Gong P., Li J., Xu N., Qin S. (2020). Morphological and molecular alterations of reactive astrocytes without proliferation in cerebral cortex of an APP/PS1 transgenic mouse model and Alzheimer’s patients. Glia.

[24] Qi G., Tang H., Gong P., Liu Y., He C., Hu J., Kang S., Chen L., Qin S. (2024). Sex-specific hypothalamic neuropathology and glucose metabolism in an amyloidosis transgenic mouse model of Alzheimer’s disease. Cell Biosci..

[25] Radde R., Bolmont T., Kaeser S.A., Coomaraswamy J., Lindau D., Stoltze L., Calhoun M.E., Jäggi F., Wolburg H., Gengler S. (2006). Aβ42-driven cerebral amyloidosis in transgenic mice reveals early and robust pathology. EMBO Rep..

[26] Qian H., Song X., He G., Peng X., Chen Y., Huang P., Zhang J., Lin X., Gao Q., Zhu S. (2025). Müller Glial-Derived Small Extracellular Vesicles Mitigate RGC Degeneration by Suppressing Microglial Activation via Cx3cl1-Cx3cr1 Signaling. Adv. Healthc. Mater..

[27] Masland Richard H. (2012). The Neuronal Organization of the Retina. Neuron.

[28] Johnson J., Tian N., Caywood M.S., Reimer R.J., Edwards R.H., Copenhagen D.R. (2003). Vesicular Neurotransmitter Transporter Expression in Developing Postnatal Rodent Retina: GABA and Glycine Precede Glutamate. J. Neurosci..

[29] Reichenbach A., Bringmann A. (2019). Glia of the human retina. Glia.

[30] Edwards M.M., Rodríguez J.J., Gutierrez-Lanza R., Yates J., Verkhratsky A., Lutty G.A. (2014). Retinal macroglia changes in a triple transgenic mouse model of Alzheimer’s disease. Exp. Eye Res..

[31] Bringmann A., Iandiev I., Pannicke T., Wurm A., Hollborn M., Wiedemann P., Osborne N.N., Reichenbach A. (2009). Cellular signaling and factors involved in Müller cell gliosis: Neuroprotective and detrimental effects. Prog. Retin. Eye Res..

[32] Nickerson P., McLeod M., Myers T., Clarke D. (2011). Effects of epidermal growth factor and erythropoietin on Müller glial activation and phenotypic plasticity in the adult mammalian retina. J. Neurosci. Res..

[33] Habiba U., Descallar J., Kreilaus F., Adhikari U.K., Kumar S., Morley J.W., Bui B.V., Koronyo-Hamaoui M., Tayebi M. (2021). Detection of retinal and blood Aβ oligomers with nanobodies. Alzheimers Dement. Diagn. Assess. Dis. Monit..

[34] Vandenabeele M., Veys L., Lemmens S., Hadoux X., Gelders G., Masin L., Serneels L., Theunis J., Saito T., Saido T.C. (2021). The AppNL-G-F mouse retina is a site for preclinical Alzheimer’s disease diagnosis and research. Acta Neuropathol. Commun..

[35] Mata N.L., Radu R.A., Clemmons R.S., Travis G.H. (2002). Isomerization and Oxidation of Vitamin A in Cone-Dominant Retinas. Neuron.

[36] Travis G.H., Golczak M., Moise A.R., Palczewski K. (2007). Diseases Caused by Defects in the Visual Cycle: Retinoids as Potential Therapeutic Agents. Annu. Rev. Pharmacol. Toxicol..

[37] Wang J.-S., Kefalov V.J. (2011). The Cone-specific visual cycle. Prog. Retin. Eye Res..

[38] Jin M., Li S., Nusinowitz S., Lloyd M., Hu J., Radu R.A., Bok D., Travis G.H. (2009). The Role of Interphotoreceptor Retinoid-Binding Protein on the Translocation of Visual Retinoids and Function of Cone Photoreceptors. J. Neurosci..

[39] Gresh J., Goletz P.W., Crouch R.K., Rohrer B. (2003). Structure–function analysis of rods and cones in juvenile, adult, and aged C57BL/6 and Balb/c mice. Vis. Neurosci..

[40] Wang W.-Y., Bin X., Xu Y., Chen S., Zhou S., Chen S., Cao Y., Qiu K., Ng T.K. (2025). The Profile of Retinal Ganglion Cell Death and Cellular Senescence in Mice with Aging. Int. J. Mol. Sci..

[41] Boccuni I., Bas-Orth C., Bruehl C., Draguhn A., Fairless R. (2023). Glutamate transporter contribution to retinal ganglion cell vulnerability in a rat model of multiple sclerosis. Neurobiol. Dis..

[42] Yao J., Yao W., Zhu J., Liu Y., Liu J., Ji Y., Ni X., Mu W., Yan B. (2024). Targeting tRNA-Derived Non-Coding RNA Alleviates Diabetes-Induced Visual Impairment through Protecting Retinal Neurovascular Unit. Adv. Sci..

[43] Shivashankar G., Lim J.C., Acosta M.L. (2021). Glyceraldehyde-3-phosphate dehydrogenase and glutamine synthetase inhibition in the presence of pro-inflammatory cytokines contribute to the metabolic imbalance of diabetic retinopathy. Exp. Eye Res..

[44] Fort P.E., Sene A., Pannicke T., Roux M.J., Forster V., Mornet D., Nudel U., Yaffe D., Reichenbach A., Sahel J.A. (2008). Kir4.1 and AQP4 associate with Dp71- and utrophin-DAPs complexes in specific and defined microdomains of Müller retinal glial cell membrane. Glia.

[45] Li J., Cao F., Yin H.L., Huang Z.J., Lin Z.T., Mao N., Sun B., Wang G. (2020). Ferroptosis: Past, present and future. Cell Death Dis..

[46] Kwon M.-Y., Park E., Lee S.-J., Chung S.W. (2015). Heme oxygenase-1 accelerates erastin-induced ferroptotic cell death. Oncotarget.

[47] Gao Y., Wang T., Cheng Y., Wu Y., Zhu L., Gu Z., Wu Y., Cai L., Wu Y., Zhang Y. (2023). Melatonin ameliorates neurological deficits through MT2/IL-33/ferritin H signaling-mediated inhibition of neuroinflammation and ferroptosis after traumatic brain injury. Free Radic. Biol. Med..

