# Supplementary material for: Early Müller Glial Activation and Retinal Ganglion Cell Synaptic Dysfunction in APP/PS1 Mice
Source: Cells. 2026 Apr 28;15(9):801. doi: 10.3390/cells15090801 (PMC13162674; doi:10.3390/cells15090801)
Supplement: Supplementary file 1 [file cells-15-00801-s001.zip › cells-4227506-supplementary.pdf]

Name	Base
**PCR**	
APP-F	GAATTCCGACATGACTCAGG
APP-R	GTTCTGCTGCATCTTGGACA
hPS1-F	CAGGTGCTATAAGGTCATCC
hPS1-R	ATCACAGCCAAGATGAGCCA
**RT-qPCR**	
Folh1-F	ATTGTGCGGAGCTTTGGAACCC
Folh1-R	AAGCCACACCTCGCTCTTGTAG
Asns-F	GCAGTGTCTGAGTGCGATGAA
Asns-R	TCTTATCGGCTGCATTCCAAAC
Slc1a4-F	GGCATCGCTGTTGCTTACTTC
Slc1a4-R	CGAGGAAAGAGTCCACTGTCT
Slc7a11-F	CTTTGTTGCCCTCTCCTGCTTC
Slc7a11-R	CAGAGGAGTGTGCTTGTGGACA
Slc3a2-F	TGATGAATGCACCCTTGTACTTG
Slc3a2-R	GCTCCCCAGTGAAAGTGGA
Socs3-F	ATGGTCACCCACAGCAAGTTT
Socs3-R	TCCAGTAGAATCCGCTCTCCT
Jun-F	CCTTCTACGACGATGCCCTC
Jun-R	GGTTCAAGGTCATGCTCTGTTT
Hmox1-F	AAGCCGAGAATGCTGAGTTCA
Hmox1-R	GCCGTGTAGATATGGTACAAGGA
Mt2-F	GCCTGCAAATGCAAACAATGC
Mt2-R	AGCTGCACTTGTCGGAAGC
Herpud1-F	CCTCCAAAATGCCAGAAACCAGC
Herpud1-R	GCCGTAAACCATCACTTGAGGAG
Aqp4-F	AGCCAGCATGAATCCAGCTCGA
Aqp4-R	TCATAAAGGGCACCTGCCAGCA
Kcnj10-F	TGCGGAAGAGTCTCCTCATTGG
Kcnj10-R	GTCTGAGGCTGTGTCTACTTGG
Cx3cr1-F	GAGTATGACGATTCTGCTGAGG
Cx3cr1-R	CAGACCGAACGTGAAGACGAG
Kdr-F	CGAGACCATTGAAGTGACTTGCC
Kdr-R	TTCCTCACCCTGCGGATAGTCA
Cwc22-F	CCAGAGACAACCCACGCAATAC
Cwc22-R	TGGTTTCTGGGCTACGATGACC
Syt12-F	GACGAGGATGAGCGGAATGTGA
Syt12-R	GGTAACTGAGGGACAGCAGGAT
S100a10-F	GACAAAGGAGGACCTGAGAGTG
S100a10-R	CTCTGGAAGCCCACTTTGCCAT
Cdkn1a-F	CCTGGTGATGTCCGACCTG
Cdkn1a-R	CCATGAGCGCATCGCAATC
Actb-F	GGCTGTATTCCCCTCCATCG
Actb-R	CCAGTTGGTAACAATGCCATGT

Figure S1

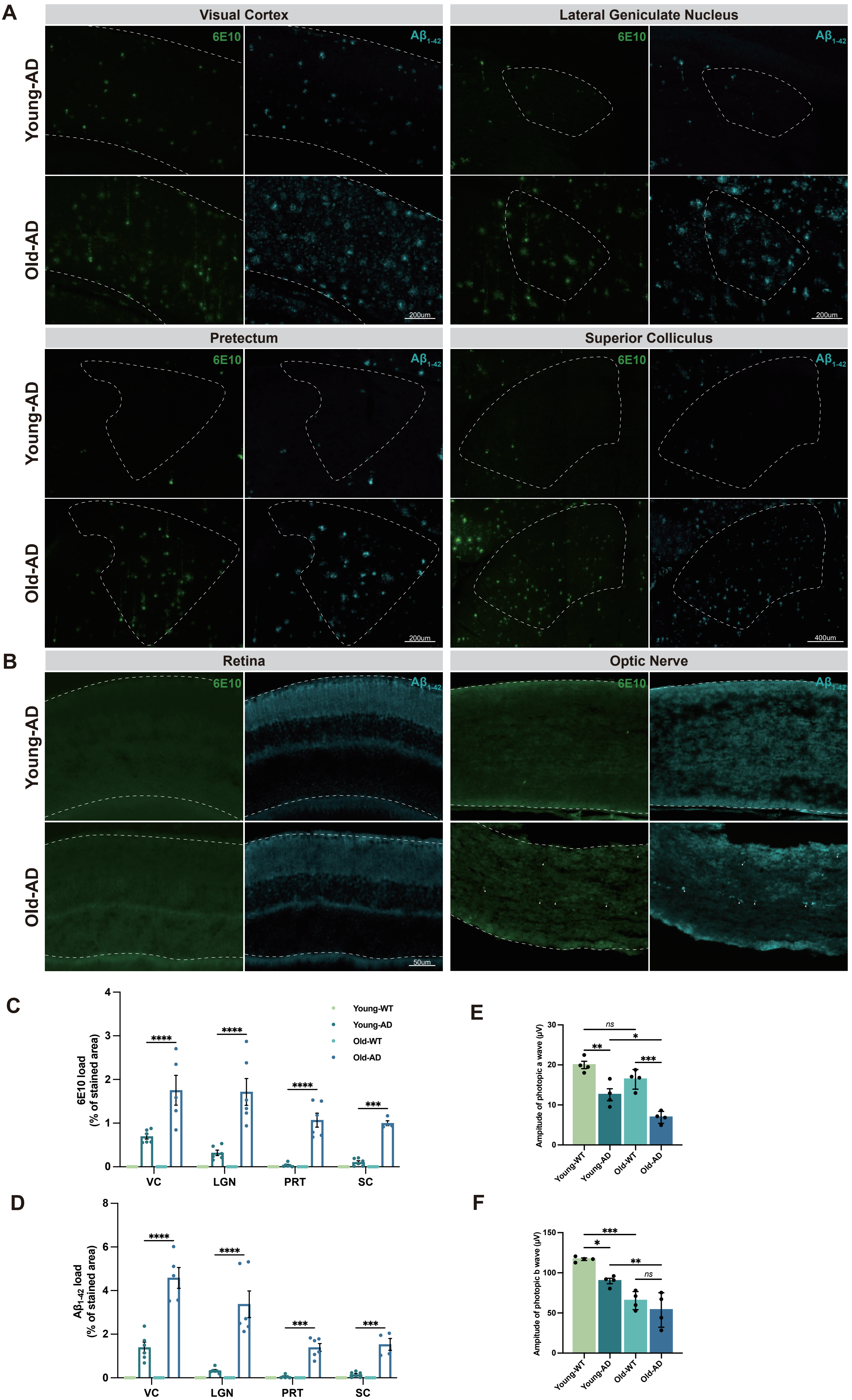
A
Visual Cortex
Lateral Geniculate Nucleus
Pretectum
Superior Colliculus
6E10
Aβ1-42
Young-AD
Old-AD
200um
400um
B
Retina
Optic Nerve
50um
C
6E10 load (% of stained area)
Young-WT
Young-AD
Old-WT
Old-AD
VC
LGN
PRT
SC
****
***
D
Aβ1-42 load (% of stained area)
E
Amplitude of photopic a wave (μV)
ns
**
*
***
F
Amplitude of photopic b wave (μV)

Figure S2

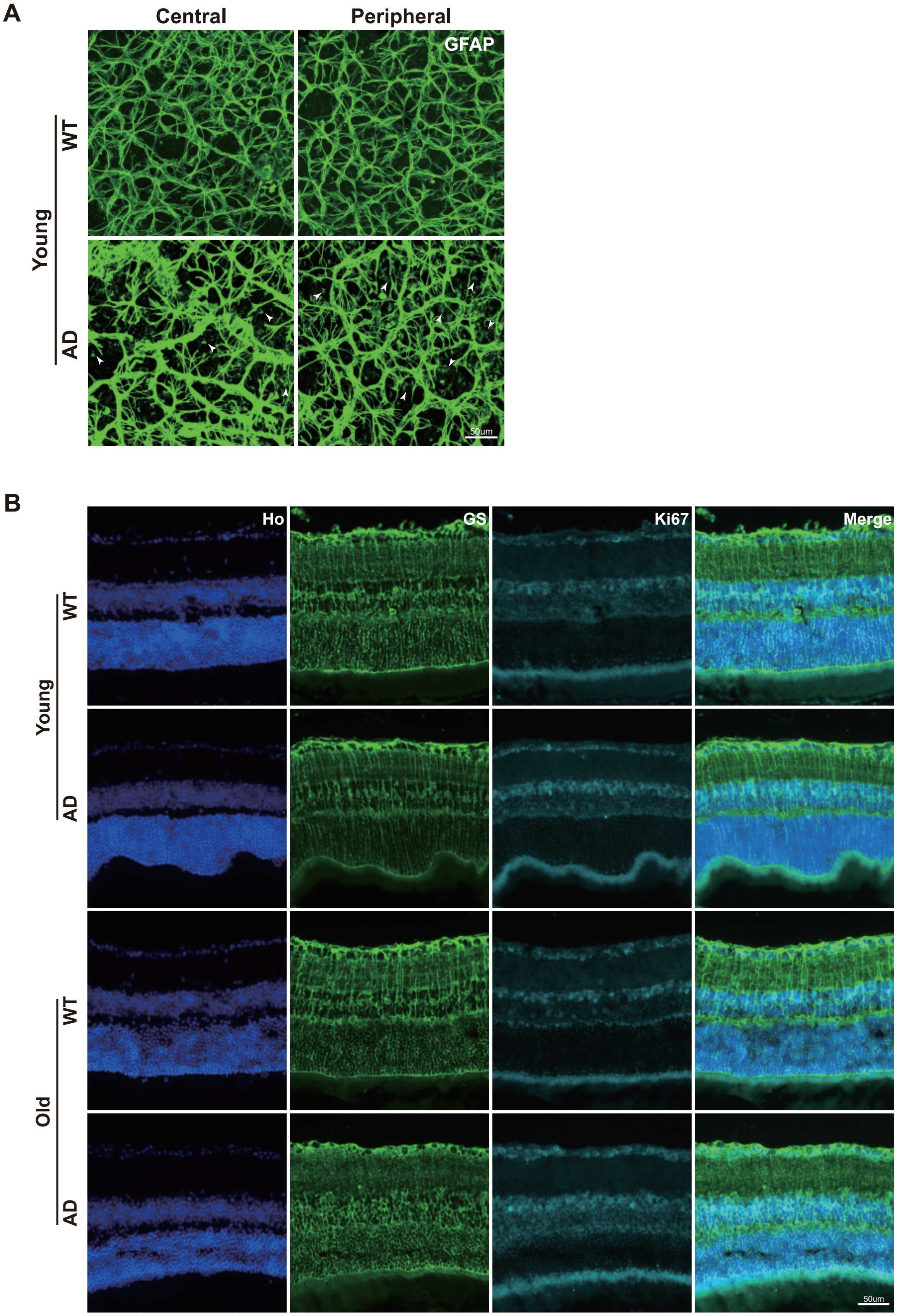
A
Central
Peripheral
GFAP
Young
WT
AD
50um
B
Ho
GS
Ki67
Merge
Young
WT
AD
Old
WT
AD
50um

Figure S3

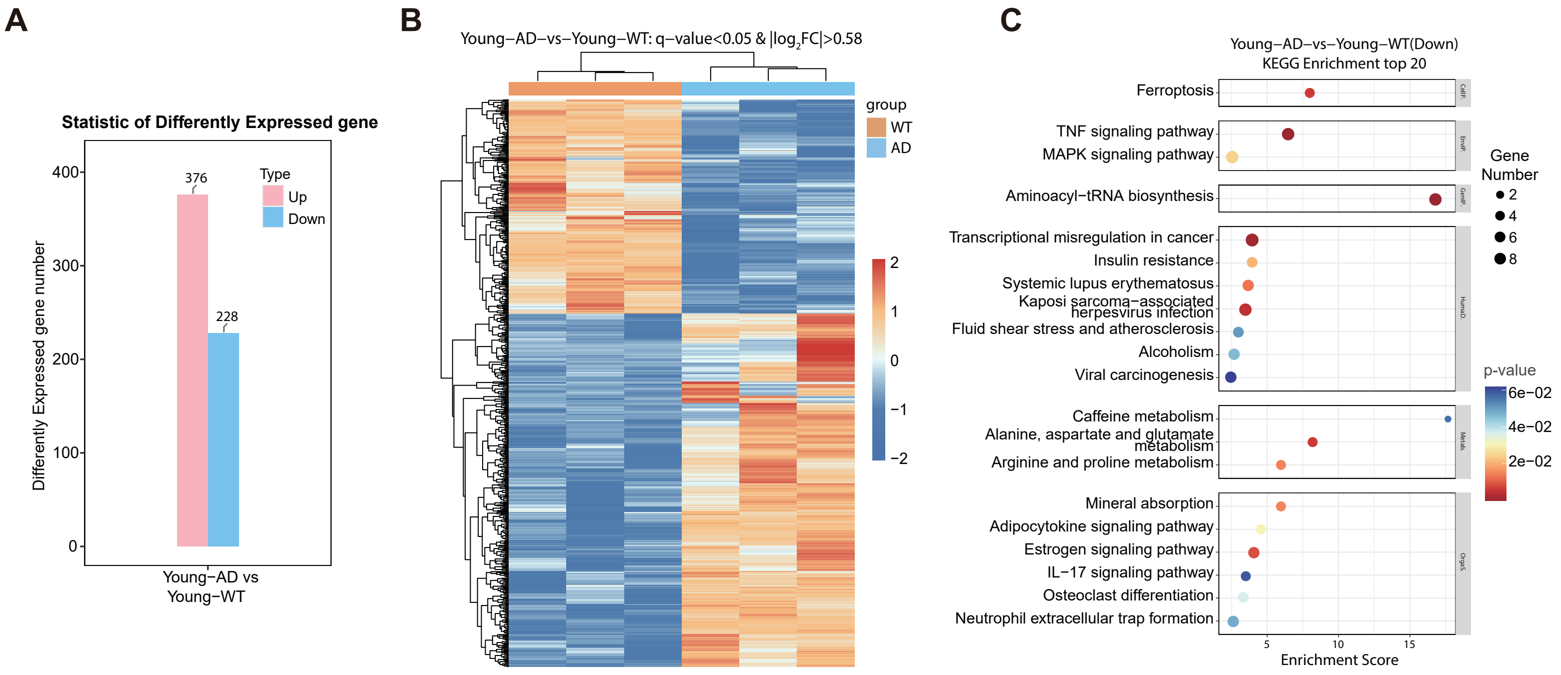
A
Statistic of Differently Expressed gene
Differently Expressed gene number
400
300
200
100
0
376
228
Type
Up
Down
Young−AD vs
Young−WT
B
Young−AD−vs−Young−WT: q−value<0.05 & |log2FC|>0.58
group
WT
AD
2
1
0
−1
−2
C
Young−AD−vs−Young−WT(Down)
KEGG Enrichment top 20
Ferroptosis
TNF signaling pathway
MAPK signaling pathway
Aminoacyl−tRNA biosynthesis
Transcriptional misregulation in cancer
Insulin resistance
Systemic lupus erythematosus
Kaposi sarcoma−associated herpesvirus infection
Fluid shear stress and atherosclerosis
Alcoholism
Viral carcinogenesis
Caffeine metabolism
Alanine, aspartate and glutamate metabolism
Arginine and proline metabolism
Mineral absorption
Adipocytokine signaling pathway
Estrogen signaling pathway
IL−17 signaling pathway
Osteoclast differentiation
Neutrophil extracellular trap formation
5
10
15
Enrichment Score
Gene Number
2
4
6
8
p-value
6e−02
4e−02
2e−02
